# Impact of biopsychosocial frailty trends on survival and quality of life of older adults: a secondary analysis of data from a community-based active monitoring program

**DOI:** 10.3389/fragi.2026.1791524

**Published:** 2026-06-26

**Authors:** Giuseppe Liotta, Olga Madaro, Clara Donnoli, Fabio Riccardi, Giulia Picardo, Fausto Ciccacci, Michele Bisogno, Paola Scarcella

**Affiliations:** 1 Biomedicine and Prevention Department, University of Rome “Tor Vergata”, Rome, Italy; 2 Community of Sant’Egidio – Long Live the Elderly Program, Rome, Italy; 3 LUMSA University, Rome, Italy

**Keywords:** assessment, biopsychosocial, frailty, older adult, survival trend

## Abstract

**Background:**

Advancing age frequently brings about a complex condition known as biopsychosocial frailty, which significantly impacts an older adult’s survival and quality of life. This research investigates how changes in frailty levels affect mortality, hospitalization, and institutionalization, utilizing data from the “Long Live the Elderly!” program, a community-based initiative focused on proactive monitoring.

**Methods:**

A secondary analysis was conducted on data from 6,802 older adults participating in the LLE program across six Italian cities. Frailty was assessed using the Short Functional Geriatric Evaluation, which encompasses physical, psychological, and socio-economic dimensions. Trends in frailty were analyzed over an average follow-up period of 4.6 years, with survival outcomes evaluated using Cox proportional hazard models.

**Results:**

At baseline, 40.0% of participants were robust, 25.9% pre-frail, 27.4% frail, and 6.7% very frail. Frailty levels deteriorated in 36.9% of participants, while 11.2% experienced improvement. Higher frailty levels were significantly associated with increased mortality, hospitalization, and institutionalization. However, individuals whose frailty remained stable or improved showed significantly lower mortality rates compared to those whose frailty declined. Socio-economic enhancements were linked to better frailty outcomes, while psychophysical deterioration was the primary driver of declines.

**Conclusion:**

Frailty is a dynamic condition that may improve or worsen over time. Stabilizing or improving frailty trajectories was associated with reduced mortality, hospitalization, and institutionalization, supporting the relevance of multidimensional frailty monitoring in community settings These findings can support the importance of proactive multidimensional frailty monitoring in aging populations.

## 1 Introduction

Biopsychosocial frailty is a multidimensional condition characterized by a decline in an individual’s physical, psychological and social capacities, which collectively increases vulnerability to adverse health outcomes as mortality, hospitalization, and institutionalization (Gobbens et al., 2010; Damiani et al., 2021). Traditionally associated with aging, frailty is increasingly recognized as a dynamic and multifactorial process rather than a static condition. The frailty biopsychosocial model underscores the interplay among biological, psychological, and social domains in shaping an individual’s frailty status. This comprehensive framework broadens the scope of understanding beyond physical decline, incorporating factors such as cognitive impairment, emotional resilience, and social support networks.

Frailty can be conceptualised as the opposite pole of healthy ageing: healthy ageing reflects the maintenance of functional ability and intrinsic capacity across biological, psychological and social domains, whereas biopsychosocial frailty represents the progressive depletion of these reserves and a higher vulnerability to stressors. Framing frailty within the healthy ageing paradigm helps clarify its public health relevance, particularly for the oldest old, in whom small changes in resources may translate into large differences in survival and quality of life (Behr et al., 2023; Silva et al., 2024). Importantly, frailty is dynamic: individuals can worsen, remain stable, or improve over time. Understanding trajectories - rather than single time-point measurements - supports risk stratification, timely preventive actions, and better allocation of community resources.

Research highlights that frailty assessed through multidimensional tools is a strong predictor of adverse outcomes (Liotta et al., 2023). Several instruments validated in literature allow multidimensional assessment of frailty. It is advisable to choose the most suitable tool for the context, purpose of the assessment, and aspect one wishes to investigate the most (Faller et al., 2019). The inclusion of social and economic variables in frailty assessment highlights the potential role of socio-economic factors—such as social isolation and lack of resources - in compounding risks. Negative outcomes often emerge from the cumulative effects of these domains, reinforcing the need for early detection and integrated interventions (Gilardi et al., 2018). Screening tools, like the Short Functional Geriatric Evaluation (SFGE), validated in its psychometric properties (Donnoli et al., 2024) incorporating socio-economic dimensions, are particularly effective in identifying individuals at higher risk and facilitating tailored care strategies (Liotta et al., 2023).

Recent advances in frailty research emphasize the importance of temporal trends in assessing and addressing this complex status (Chiu et al., 2022; Tchalla et al., 2023). Shifts in demographic patterns, healthcare interventions, and societal structures have significant implications in the prevalence and progression of frailty across populations Newman-Norlund et al., 2022. Investigating these trends provides critical insights in the evolving landscape of frailty and informs the development of tailored interventions aimed at mitigating its impact. Understanding trajectories of biopsychosocial frailty is particularly relevant in community-dwelling older populations, where social and economic vulnerabilities frequently coexist with functional decline. Longitudinal evaluation of multidimensional frailty may improve risk stratification and support early identification of individuals at higher risk of adverse outcomes. However, evidence describing changes in biopsychosocial frailty over time and their association with mortality, hospitalization, and institutionalization in real-world community programs remains limited (Chiu et al., 2022; Tchalla et al., 2023; Liu et al., 2024; De Luca et al., 2022).

This study aims to investigate trajectories of biopsychosocial frailty over time in older adults enrolled in a community-based active monitoring program and to assess their association with adverse outcomes. Specifically, we sought to: (1) describe changes in frailty status over time; and (2) evaluate the association between frailty trajectories (improvement, stability, or worsening) and mortality, hospitalization, and institutionalization. By adopting a biopsychosocial perspective, this study provides insight into how multidimensional frailty evolves in real-world community settings and how different frailty trajectories relate to clinically relevant outcomes.

## 2 Methods

This study is a secondary analysis of prospectively collected data from the “Long Live the Elderly!” (LLE) program, a community-based proactive monitoring program for older adults. The analysis was conducted as a retrospective cohort study aimed at evaluating trajectories of biopsychosocial frailty over time and their association with adverse outcomes, including mortality, hospitalization, and institutionalization. Participants were followed longitudinally from their first available frailty assessment until the occurrence of an outcome event or censoring at the end of the observation period.

### 2.1 “The long live the elderly!” program

The “Long Live the Elderly!” (LLE) program is a community-based proactive monitoring initiative implemented by the Community of Sant’Egidio, in collaboration with local municipalities. The program aims to identify and monitor older adults living in the community and to reduce social isolation and vulnerability through individualized support. The program primarily targets residents aged 80 years and older, identified using municipal registries. Individuals aged 75 years and older may also voluntarily enroll. After initial contact, participants provide consent and are included in the monitoring program. Biopsychosocial frailty is assessed at baseline and annually using the Short Functional Geriatric Evaluation (SFGE) (Liotta et al., 2023), administered by trained program operators. However, the frequency and timing of follow-up assessments were not fixed across participants, as monitoring intensity varied according to individual needs and frailty level within the program. Based on the assessment results, participants receive individualized follow-up, including telephone monitoring, activation of formal services, and involvement of informal support networks when needed. The frequency of contacts varies according to frailty level, ranging from periodic phone calls to more frequent monitoring. Operators receive standardized training and work under the supervision of social workers responsible for program coordination. Although assessment procedures are standardized, interventions are personalized according to individual needs. Therefore, the program does not represent a uniform intervention but rather a structured monitoring framework with tailored support strategies. Information on adverse outcomes, including hospitalization, institutionalization, and death, is collected during routine follow-up contacts and updated periodically in the program database.

### 2.2 Selection of participants’ records to be included in the analysis

The initial database included individuals enrolled in the LLE program in six Italian cities (Brindisi, Genoa, Naples, Novara, Rome and Sassari) where the program had been operating for at least 5 years between 1 January 2016 and 24 July 2024. This time frame was selected to ensure sufficient program maturity and availability of longitudinal assessments.

Participants were eligible for inclusion if they: (1) were aged 75 years or older; (2) had at least two assessments of biopsychosocial frailty using the SFGE; and (3) had a minimum follow-up of 180 days.

Participants with missing data on key variables required for the analysis were excluded from the final analytical sample.

For each participant, baseline was defined as the first available SFGE assessment, and follow-up extended until the last available assessment or the occurrence of an outcome event (death, hospitalization, or institutionalization), whichever came first. Participants without outcome events were censored at the end of the observation period.

The observed minimum age of 74 reflects a small number of participants who were 74 years old at the time of initial contact but turned 75 at the first annual assessment, thereby meeting the program inclusion criteria.

A flowchart describing participant selection, exclusions, and final sample size is provided in Supplementary Material S1.

### 2.3 Ethics statements

The anonymized data used for the secondary analysis conducted in this study are the result of interviews conducted by LLE program staff following the obtainment of the informed consent, agreed upon with each municipality involved in the program. The use of the collected variables was evaluated and approved by the ethical committee (Ethical Committee of the University of Rome Tor Vergata R.S. 60/17).

### 2.4 Measures

The main exposure was frailty trajectory, defined according to changes in biopsychosocial frailty status between the first and last available SFGE assessments. Participants were classified as “improved” if their final frailty level was less severe than the initial one, “stable” if it remained unchanged, and “worsened” if the final level was more severe than the initial one.

Biopsychosocial frailty was assessed using the Short Functional Geriatric Evaluation (SFGE), a 12-item multidimensional tool administered by trained program staff. This questionnaire is a brief multidimensional tool administered by trained program staff. It includes 12 items organized into two domains: (i) socio-economic and social vulnerability (items 1–7: age, education, living arrangement, availability of informal social support, access to formal municipal or health services, and economic difficulties); and (ii) psychophysical and functional domains (items 8–12: psychological/motivational status, ability to bathe independently, ability to leave home, functional autonomy, and overall health status). Each item is scored and summed to obtain a total score that is then categorised into four frailty levels: Robust (score ≤0), Pre-frail (1–2), Frail (3–9), and Very frail (≥10), as previously described and validated (Liotta et al., 2023; Donnoli et al., 2024).

Covariates included age group, gender, hospitalization during the observation period, years of program operation, and city of enrollment. These variables were selected based on clinical relevance and availability in the dataset.

Outcomes were mortality, hospitalization, and institutionalization. Mortality was defined as the death date reported by family members or caregivers. Hospitalization was defined as any acute hospital admission during the observation period. Institutionalization was defined as permanent relocation to a long-term care facility. According to the program follow up protocol this information was either requested every 6 months from the subjects or from people close to them (relatives or caregivers) or recorded in the database when learned during routine telephone monitoring or because spontaneously communicated by the subjects or people close to them.

### 2.5 Statistical analysis

To explore the evolution of biopsychosocial frailty over time and its impact on clinical outcomes, we adopted a sequential analytical approach consisting of three main steps: (1) description of the sample and frailty distribution at baseline; (2) analysis of changes in frailty status between first and last available assessment; and (3) evaluation of the association between frailty trajectories and adverse outcomes (mortality, hospitalization, and institutionalization) using survival analysis.

The sample was described through the frequency, distribution and mean of the variables, as appropriate.

Kruskal-Wallis test was used to compare the means of more than two samples, while the Wilcoxon test was used to compare the distributions in two correlated observations with non-normal distribution on the same sample. The Chi-square test was used to compare categorical variables. The Mann Whitney U test was used to compare non-normal distributions in two independent samples. Pearson correlation was used to assess the relationship between changes in socio-economic and psychophysical domains. Inferential analysis was performed by multivariable Cox proportional hazards regression to estimate the association between frailty trajectories and mortality. The model included the following covariates: frailty trajectory (as the main exposure, with Very Frail worsened as the reference category), age group, gender, hospitalization during the observation period (as a proxy for psychophysical impairment severity), years of program operation, and city of enrollment. Variables were selected based on their clinical relevance and availability in the dataset.

The p-value less than 0.05 was considered an indicator of statistical significance. Statistical analyses were conducted using the SPSS Statistics software v.26.

## 3 Results

The initial database comprised 10,427 records, of which 3,625 were excluded due to inclusion criteria. In the analysis 6,802 participants were included and distributed as it follows: mean participant age was 83.8 (SD ± 4.87; range 74–106), 65.9% were women. Mean observation time was 4.8 years (SD ± 1.5; range 0.5–8.0). Biopsychosocial frailty assessment results from the first SFGE questionnaire are in Table 1.

**TABLE 1:** Sample description.

Biopsychosocial frailty first assessment	Robust N = 2,720 (40.0%)	Pre-frail N = 1,760 (25.9%)	Frail N = 1,862 (27.4%)	Very frail N = 460 (6.7%)	Overall N = 6,802 (100.0%)
Characteristic					
Age (years)^1^	82.2 (4.18)	84.4 (4.57)	85.0 (5.15)	86.7 (5.36)	83.8 (4.87)
Years of observation^1^	5.06 (1.36)	4.83 (1.41)	4.55 (1.54)	3.86 (1.57)	4.78 (1.47)
Origin^2^					
Brindisi	43 (1.6%)	49 (2.7%)	72 (3.8%)	26 (5.7%)	190 (2.7%)
Genova	136 (6.8%)	136 (7.7%)	169 (9.0%)	26 (5.7%)	515 (7.5%)
Napoli	606 (22.3%)	455 (25.9%)	639 (34.5%)	151 (32.8%)	1,851 (27.3%)
Novara	386 (14.2%)	294 (16.8%)	186 (10.0%)	31 (6.7%)	897 (13.2%)
Roma	1,411 (51.8%)	757 (43,0%)	747 (40.1%)	209 (45.4%)	3,124 (46.1%)
Sassari	90 (3.3%)	69 (3.9%)	49 (2.6%)	17 (3.7%)	225 (3.2%)
Death (N)	653	562	870	307	2,392
Hospitalization (Cumulative N of)	1,152	809	1,211	240	3,420
Institutionalization (N)	72	101	130	37	340

1 Mean (SD).

2 n. (%).

During observation the percentage of robust individuals decreased from 40% to 24%, while very frail individuals increased from 6.8% to 14.2% (Wilcoxon Test: p < 0.001 – Table 2). Frailty status worsened for 36.9% of the participants (sum above diagonal), while improved for 11.2% of them (sum below diagonal), and remained unchanged for 51.9%.

**TABLE 2:** Distribution of biopsychosocial frailty according to the timing of assessment.

Categories		Final assessment				
		Robust	Pre-frail	Frail	Very frail	Total
First assessment	Robust (n. 2,720)	1,316	723	529	152	2,720
		19.30%	10.60%	7.80%	2.20%	40.00%
	Pre-frail (n. 1,760)	201	725	674	160	1,760
		3.00%	10.70%	9.90%	2.40%	25.90%
	Frail (n. 1,862)	100	309	1,110	343	1,862
		1.50%	4.50%	16.30%	5.00%	27.40%
	Very frail (n. 460)	13	25	110	312	460
		0.20%	0.40%	1.60%	4.60%	6.80%
	Total (n. 6,802)	1,630	1,782	2,423	967	6,802
		24.00%	26.20%	35.60%	14.20%	100.00%

Improvement was higher among frail (409/1862–21.9%) and pre-frail (201/1760–11.4%), while robust individuals experienced most frequently worsening (1,404/2,720%–51.6%). Those who reduced frailty, improved mainly socio-economic condition (71.1% improved in items 3–7) (Supplementary S2): No. 3 (Living arrangement), No. 4 (Having someone to rely on) and No. 7 (Economic status) were the most improved items. Participants who increased frailty worsened mainly in psychophysical items (60.8% worsened in items 8–12) (Supplementary S3): No. 8 (Psychological status), No.9 (Being able to bath/shower independently) and No. 10 (Being able to leave home) were the most worsened items. An inverse correlation was observed between changes in socio-economic and psychophysical items (Pearson correlation = −0.129; p < 0.001), indicating a weak association at the group level.

To understand the impact of improving frailty status, each initial frailty category was divided in two strata: participants who reduced frailty or remained at the same level vs. those who increased their frailty level (Table 3). Analysis of mortality, hospitalization, and institutionalization rates shows statistically significant differences, within the same strata of initial frailty, between those who did not deteriorate (stable or improved frailty) and those who did deteriorate (worsened frailty) except for hospitalization and institutionalization in the “Very Frail” strata (Table 3). Overall, participants with stable or improved frailty trajectories showed lower hospitalization rates (138‰ vs. 112‰) and lower institutionalization rates (21‰ vs. 14‰) over 5 years compared to those with worsening frailty trajectories (Table 3).

**TABLE 3:** Death, hospitalization and institutionalization rates per 1,000 p/y according to the frailty trend.

Categories		N	Mean per 1,000 p/Y	95% CI			
				Lower limit	Upper limit	p^1^	p^2^
Death	Robust stable	1,348	69.1	60	78	0.004	<0.001
	Robust worsened	1,372	73.4	66	81		
	Pre-frail stable-improved	960	85.4	75	95	0.002	
	Pre-frail worsened	800	105.2	93	117		
	Frail stable-improved	1,534	133.3	124	143	<0.001	
	Frail worsened	328	210.4	189	232		
	Very frail improved	143	166.3	132	200	<0.001	
	Very frail stable	317	267.9	242	294		
Hospitalization	Robust stable	1,348	72.2	61	83	<0.001	<0.001
	Robust worsened	1,372	115.2	101	129		
	Pre-frail stable-improved	960	95.7	80	111	0.001	
	Pre-frail worsened	800	124.2	106	143		
	Frail stable-improved	1,534	153.5	135	172	0.027	
	Frail worsened	328	245.5	186	305		
	Very frail improved	143	165.5	196	225	0.461	
	Very frail stable	317	156.2	111	201		
Institutionalization	Robust stable	1,348	4.3	2	6	<0.001	<0.001
	Robust worsened	1,372	11.7	8	16		
	Pre-frail stable-improved	960	11.8	8	16	0.016	
	Pre-frail worsened	800	22.0	16	28		
	Frail stable-improved	1,534	22.3	17	28	0.011	
	Frail worsened	328	42.5	27	58		
	Very frail improved	143	29.2	9	50	0.556	
	Very frail stable	317	38.1	22	54		

1 U-Mann Withney test.

2 Kruskal-Wallis test.

**FIGURE 1:**
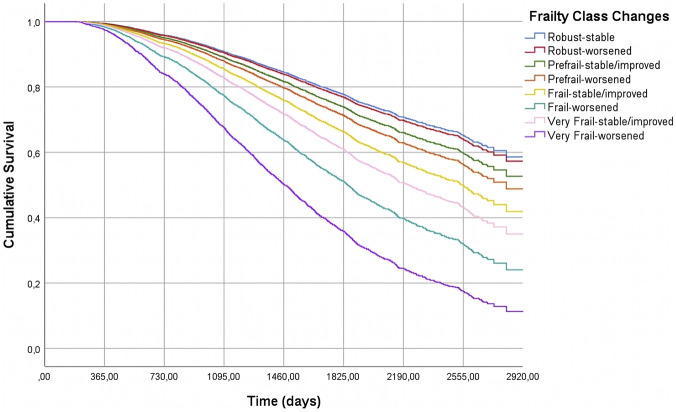
Cumulative survival according to the trend of frailty; Cox Proportional Risk analysis (p < 0.001).

Figure 1; Table 4 show survival analysis results for frailty trends using Cox proportional risk model, adjusted for age, gender and hospital admission. Hospitalization frequency serves as a proxy for psycho-physical impairment severity. The Cox model was selected for mortality only because exact event dates were available for deaths, whereas precise dates were not systematically available for hospitalization and institutionalization events, precluding reliable time-to-event analysis for those outcomes. The analysis also included program implementation years for each city since they relate to operator experience and territorial network extension and city of enrollment to account for contextual differences related to the different city size and/or location of the urban zone where the LLE program operates and/or public community service provision. Figure 1 shows that cumulative survival varies with frailty trends. The results show a clear gradient of mortality risk across frailty trajectories. Compared to Very Frail individuals who worsened (reference category), all other trajectory groups showed significantly lower mortality risk (p < 0.001), with Robust–stable participants having the lowest hazard ratio (HR = 0.25). Notably, even Very Frail individuals who stabilized showed a substantially lower mortality risk (HR = 0.48) compared to those who worsened, suggesting that stabilization of frailty status is associated with lower mortality risk regardless of baseline frailty level. Among the frail and very frail, stabilizing or improving frailty was associated with approximately 30% lower mortality compared to worsening. Higher age, male sex, and previous hospitalization were also independently associated with increased mortality risk (Table 4).

**TABLE 4:** Cox Proportional risk analysis for death.

Variables	Sign	HR	95,0% CI	
			Lower	Upper
Age (<85, 85–95, >95)	<0.001	1.91	1.76	2.08
Admission (yes vs. no)	<0.001	1.91	1.75	2.10
Gender (male vs. female)	<0.001	1.50	1.38	1.63
LLE years of operation	<0.001	0.85	0.79	0.93
Very Frail – worsened (ref.)	​	1.00	​	​
Robust - stable	<0.001	0.25	0.20	0.29
Robust - worsened	<0.001	0.26	0.22	0.30
Prefrail - stable/improved	<0.001	0.29	0.25	0.35
Prefrail - worsened	<0.001	0.33	0.28	0.39
Frail - stable/improved	<0.001	0.40	0.34	0.47
Frail - worsened	<0.001	0.65	0.54	0.79
Very frail - stable/improved	<0.001	0.48	0.37	0.63
Rome (ref.)	​	1.00	​	​
Brindisi	<0.001	2.73	2.03	3.64
Genoa	<0.001	1.35	1.14	1.61
Naples	<0.001	1.67	1.51	1.85
Novara	0.538	1.06	0.92	1.22
Sassari	0.082	1.24	0.97	1.60

**FIGURE 2:**
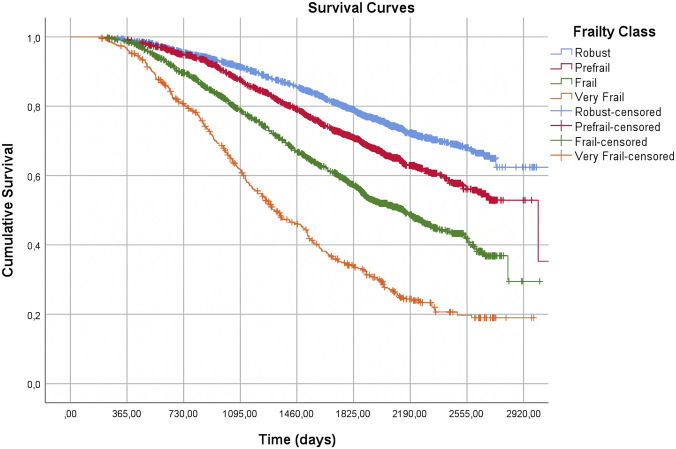
Kaplan-Meier survival curves stratified by baseline frailty level.

Figure 2 shows the Kaplan-Meier survival curves stratified by baseline frailty level. Cumulative survival differed significantly across the four groups, with Robust participants showing the highest survival probability throughout the observation period, followed by Pre-frail and Frail participants. Very Frail individuals at baseline showed the steepest decline in cumulative survival.

## 4 Discussion

This study examined trajectories of biopsychosocial frailty in a large community-based cohort of older adults and their association with mortality, hospitalization, and institutionalization.

Biopsychosocial frailty arises from physical, psychological, and socio-economic vulnerabilities, making interventions require a holistic approach (Cohen et al., 2023; Lozupone et al., 2024). It evolves over time, influenced by individual and contextual factors (Barghouth et al., 2024). The SFGE derived biopsychosocial frailty score functions as a composite measure integrating physical health, comorbidity, cognitive status, and socio-economic vulnerability. While traditional models adjust for multiple confounders, the frailty construct’s multidimensional nature encapsulates several domains, serving as a proxy for overall vulnerability.

Managing frailty at the community level may help limit adverse effects on individuals and healthcare systems. However, community intervention is often sectorial and focused on physical independence but lacking integration between social and health components. Recent systematic reviews addressed preventing psycho-physical decline in older adults indicate multi-sector intervention based on Individualized Care Plan are potentially the most effective, though results are not conclusive (Crocker et al., 2024; Palapar et al., 2024). Social isolation interventions are typically separate from psychophysical disability efforts, though frailty is multidimensional (Adekpedjou et al., 2023). Kastner and colleagues showed social intervention’s impact on reducing social frailty, but results are not linked to healthcare service use (Kastner et al., 2024).

Regression of physical frailty in Pre-Frail individuals has been observed (Teh et al., 2022; Luo et al., 2024) while regression of Biopsychosocial frailty lacks sufficient study. Studies on frailty trajectories rarely examine both social and health components (Guo et al., 2024). When studied together, worsening social frailty correlates with physical decline (Liu et al., 2024). Our case study shows inverse correlation between psycho-physical decline and increased socio-economic resources. Individual trajectories of physical decline are followed by increased social and economic support to limit the impact on quality of life within community-based support programs. In the sample, either due to minimal physical decline or intense social-economic support, these processes balance, resulting often in stable or improved biopsychosocial frailty.

Differences in mortality risk across cities (Table 4) reflect the varying degree of program maturity and local context. Rome, as the reference category, represents the most established program site with the longest operational experience and the most developed territorial network. Cities such as Brindisi (HR = 2.73) and Naples (HR = 1.67) show higher mortality risk compared to Rome, which may reflect both shorter program implementation history and differences in the availability and integration of community social and healthcare services across urban contexts.

The central question is whether reducing biopsychosocial frailty correlates with reduced frailty health consequences. Specifically, if assessing biopsychosocial frailty at community level and implementing interventions to improve it can change mortality, hospitalization or institutionalization rates (Hikichi et al., 2015). In this observational study, stratification by initial biopsychosocial frailty and identification of two trajectories for each frailty level revealed significant differences in adverse event incidence as it is in other condition (Emberti Gialloreti et al., 2018). These findings have relevant implications: the association between frailty trajectory and adverse outcomes suggests that identifying and monitoring biopsychosocial frailty at community level may support better targeting of care resources, potentially contributing to improved outcomes and more sustainable healthcare utilization in aging populations.

Due to uncertainty in international literature, a structured approach to frailty management remains undeveloped (Sadler et al., 2018; Briggs et al., 2022). While a biopsychosocial frailty management model at community level is not defined, the evidence of its effectiveness is emerging (Yu et al., 2020; Teh et al., 2022; Woo, 2022). These findings support the potential value of complementing disease-oriented approaches with multidimensional biopsychosocial frailty assessment in older adults’ care. For example, along with keeping record of the number of diabetic patients, we should know how many require care, live alone, cannot manage their diet, or lack support. These factors are components of biopsychosocial frailty and determine population health demands. This approach does not neglect treating pathologies but recognizes that multimorbidity and its links with socio-economic resources shape older population care needs (Gobbens et al., 2010). In conclusion, this real-world longitudinal analysis suggests that stabilising or improving biopsychosocial frailty—particularly by addressing social and economic vulnerabilities—is associated with better outcomes. Given the observational design and the fact that interventions were not standardised nor randomised, these findings should not be interpreted as evidence of a program effect; rather, they support the relevance of biopsychosocial frailty monitoring and within community care pathways.

## 5 Limitations

This work has several limitations. This study is based on a secondary analysis of programmatic data, meaning participants were not randomly selected and received tailored interventions within the program. Therefore, while we observed improvements in frailty status and associated outcomes, these findings may not be solely attributable to the intervention. However, the correlation between frailty reduction and fewer adverse events is consistent with broader literature, suggesting that lowering frailty—regardless of the mechanism - remains a critical objective in community-based elder care.

Another important limitation regards the empirical process for detecting negative events such as death, hospitalisation and institutionalisation: information is collected from participants or their relatives. In some cases, it was not possible to receive information on people who suddenly disappeared, which may have led us to underestimate the frequency of negative events. However, this was possible for both groups (improved/stable and worsened) so as not to influence the analysis. The second limitation is that the presented data are the result of a secondary analysis of the information collected during the day-to-day running of the program, which means that important elements may have been missed. In fact, the frequency of telephone monitoring varies by level of frailty, from one call every 3 months for the “Robust” to one every week or even more for the “Very Frail”; again, this should not affect the analysis because the comparison was made within the same level of frailty among individuals who were offered the same protocols.

An additional methodological consideration concerns the temporal relationship between frailty trajectory classification and outcome occurrence. Frailty trajectories were defined using the first and last available SFGE assessments, while mortality outcomes were recorded throughout follow-up. This may introduce a potential survivor or immortal time bias, as participants needed to survive long enough to undergo repeated assessments. However, within the LLE program, participants were routinely monitored through scheduled contacts approximately every 6 months, and mortality information was continuously updated during follow-up. Furthermore, comparisons were performed within baseline frailty strata, partially limiting heterogeneity in follow-up intensity and risk profile.

A further limitation of this study is the lack of clinical data that, of course, affect the trends we described. Furthermore, the classification of frailty change into three categories (improved, stable, worsened) does not capture the magnitude of transitions: a change from Robust to Pre-frail and from Robust to Very frail is both classified as “worsened.” This approach, while more reliable given the category-level validation of the SFGE, necessarily reduces the granularity of the information on individual trajectories.

The program-based nature of the sample represents an additional source of potential selection bias: participants were not randomly selected from the general population but were identified through municipal registries and voluntarily enrolled. Individuals who agreed to participate may differ systematically from those who declined or were unreachable, potentially being more socially connected or less severely frail. This limits the generalizability of the findings to the broader older adult population. Despite these limitations, the value of the data presented remains relevant.

## 6 Conclusion

Biopsychosocial frailty is progressively becoming a crucial synthetic indicator of the older adults’ population need for care. Frailty is a dynamic status that may improve or worsen over time and stable or improved frailty trajectories were associated with significantly lower mortality rates. While our findings confirm the association between frailty improvement and reduced adverse outcomes, the observational nature of the study precludes definitive conclusions regarding causality. Older adults with stable or improved frailty trajectories showed more favorable outcomes, suggesting that multidimensional monitoring of social, economic, and psychophysical vulnerabilities may help identify individuals at lower risk of adverse events. Moreover, given the observed association between stable or improved frailty trajectories and lower rates of mortality, hospitalization, and institutionalization, these findings suggest that supporting frailty stabilization may contribute to reduced healthcare utilization, although direct evidence on costs and quality of life remains to be established in future studies. While community programs offer substantial benefits, challenges remain in scaling these initiatives to reach wider populations. Ensuring consistent funding, training personnel, and integrating services across sectors are critical to their sustainability. Future efforts should focus on expanding these models, leveraging technology for remote monitoring, and conducting longitudinal studies to further refine their effectiveness.

## Data Availability

The original contributions presented in the study are included in the article/Supplementary Material, further inquiries can be directed to the corresponding author.
