# Supplementary material for: Impact of biopsychosocial frailty trends on survival and quality of life of older adults: a secondary analysis of data from a community-based active monitoring program
Source: Front Aging. 2026 Jun 26;7:1791524. doi: 10.3389/fragi.2026.1791524 (PMC13350052; doi:10.3389/fragi.2026.1791524)
Supplement: Supplementary file 1 [file Supplementaryfile1.docx]

Supplementary Material

# Supplementary Figures and Tables


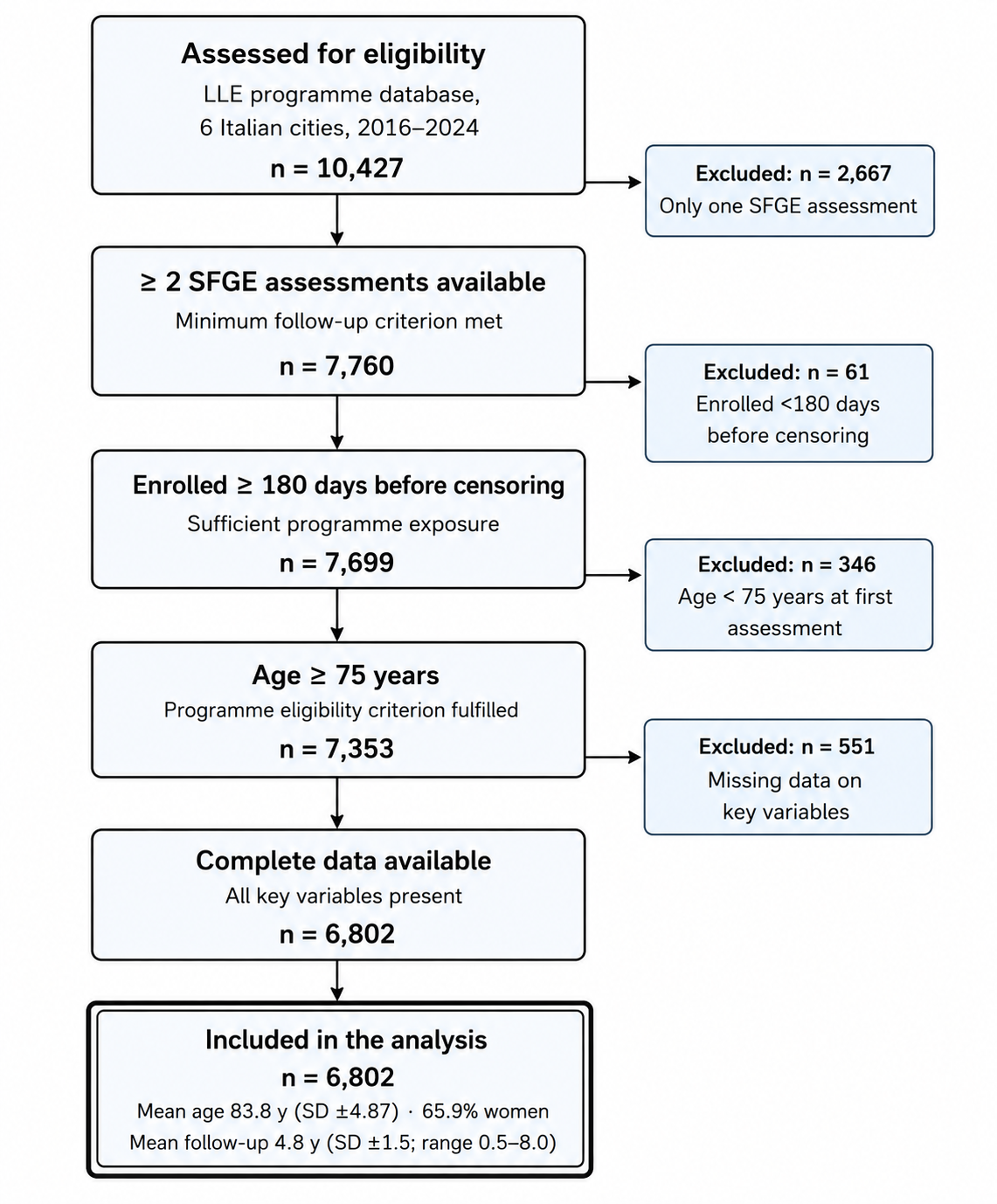


*S1. Participants selection flowchart*

*S2. Sub-sample of participants who reduced frailty during the observation according to the SFGE items*

*S3. Sub-sample of participants who increased frailty during the observation according to the SFGE items*
